# Does Support Meet the Need? A Focus Group Study on Parental Support and Students’ Psychological Need Satisfaction in a Minority School Context

**DOI:** 10.3390/healthcare14081082

**Published:** 2026-04-18

**Authors:** Aikaterini Vasiou, Servet Altan, Eleni Vasilaki, Aristea Mavrogianni, Georgios Vleioras, Marinos Anastasakis, Konstantinos Mastrothanasis

**Affiliations:** 1 Department of Primary Education, University of Crete, 74100 Rethymno, Greece; vasilaki@uoc.gr (E.V.); amavrog@uoc.gr (A.M.); m.anastasakis@uoc.gr (M.A.); 2 Psychological Guidance and Counseling Department, MEF University, İstanbul 34396, Türkiye; altans@mef.edu.tr; 3 Department of Primary Education, University of Thessaly, 38221 Volos, Greece; vleioras@uth.gr; 4 School of Medicine, National and Kapodistrian University of Athens, 11527 Athens, Greece; kmastroth@med.uoa.gr

**Keywords:** basic psychological need satisfaction, intersectionality, minority community, parental support, resilience

## Abstract

**Highlights:**

**What are the main findings?**

- Parental support contributes to minority students’ satisfaction of autonomy, competence, and relatedness.
- Minority educational contexts shape students’ psychological well-being through sociocultural and institutional conditions.

**What are the implications of the main findings?**

- Supportive parenting acts as a protective factor that promotes resilience and psychological well-being among minority students.
- School and community interventions should address the psychosocial needs of bilingual minority students.

**Abstract:**

**Background:** Parental practices that support autonomy, provide structure, and foster warm relationships are associated with greater satisfaction of students’ basic psychological needs for autonomy, competence, and relatedness. In minority educational contexts, however, students’ psychological need satisfaction is also shaped by broader sociocultural conditions that may create additional pressures and sources of chronic stress. Within such environments, parental support may function as a protective factor that helps students cope with educational and cultural demands. **Objective:** The aim of this study was to explore how parental support contributes to the satisfaction of students’ basic psychological needs within a minority educational context where students from the Greek minority attend a bilingual school operating within a Turkish educational framework. **Methods:** A qualitative design was employed using three focus groups conducted in a minority school located in Gökçeada, Türkiye: one with parents (N = 5), one with lower secondary school students (N = 6), and one with upper secondary school students (N = 6). Interview questions were developed on the basis of Basic Psychological Needs Theory. Data were analyzed thematically by five members of the research team. **Results:** Findings indicated that parental support influenced students’ need satisfaction through practices related to autonomy (e.g., trust, space for mistakes), competence (e.g., encouragement, comparison), and relatedness (e.g., emotional presence, empathy). However, these practices were not experienced in a uniform way. Rather, their meaning and impact were shaped by contextual conditions associated with minority status, including bilingual educational demands, limited resources, and close-knit community dynamics. **Conclusions:** The study suggests that in minority school settings, parental support operates not simply as a general interpersonal resource but as a contextually mediated protective process. By showing how sociocultural and institutional conditions shape the enactment and experience of autonomy, competence, and relatedness, the findings extend existing BPNT research beyond majority settings and offer a more context-sensitive understanding of students’ psychological need satisfaction.

## 1. Introduction

The satisfaction of students’ basic psychological needs has been consistently associated with positive developmental and educational outcomes, including well-being, engagement, and academic adjustment [1]. The present study is primarily grounded in Basic Psychological Needs Theory (BPNT), within which the role of significant social agents (particularly parents) has been highlighted as crucial in supporting or frustrating children’s psychological functioning [2]. Parental practices that foster autonomy, competence, and relatedness are considered essential for promoting adaptive outcomes across developmental stages (e.g., Soenens et al. [2]; Vansteenkiste et al. [3]).

At the same time, increasing attention has been given to the ways in which sociocultural contexts shape the provision and experience of such support [4]. Students growing up in minority contexts often face additional challenges, including language barriers, institutional constraints, and cultural tensions, which may influence both parental practices and children’s psychological need satisfaction (e.g., Chen et al. [5]).

To further contextualize these experiences, intersectionality theory [6,7] is used as a complementary perspective, suggesting that multiple identity dimensions, such as ethnicity, language, and minority status, interact with environmental factors and influence individuals’ opportunities and challenges. These dimensions may complicate the ways in which support is expressed, perceived, and internalized within families.

Despite the growing body of research on parental support and psychological need satisfaction, most existing studies have focused on majority populations and have primarily employed quantitative methodologies. As a result, there is limited understanding of how parental support is experienced and interpreted by students and parents in minority educational settings, particularly through in-depth qualitative approaches that can capture the complexity of everyday interactions and contextual influences.

To address this gap, the present study aims to explore how parental support contributes to students’ psychological need satisfaction within a minority context. Specifically, the study seeks to examine how both students and parents perceive and describe supportive practices, and how these are shaped by the broader sociocultural environment.

Notably, the study differs from prior research in three important ways. First, it adopts a qualitative approach, allowing for an in-depth exploration of how parental support is experienced and interpreted by both students and parents. Second, it focuses on a minority bilingual school context, where students navigate multiple linguistic and cultural demands. Third, it integrates BPNS with an intersectionality-informed perspective, to capture how psychological need satisfaction is shaped by overlapping sociocultural conditions.

The study is guided by the following research questions:

1. How do parents support students in satisfying their basic psychological needs?
2. Are there any barriers and facilitators of students’ psychological need satisfaction?
3. Does the minority status satisfy or frustrate students’ basic psychological needs?

From a healthcare perspective, understanding the familial and social factors that support or hinder these processes is essential for developing preventive and supportive interventions, particularly in vulnerable populations such as minority students. Empirical research grounded in BPNS demonstrates that the satisfaction of autonomy, competence, and relatedness is positively associated with both psychological and physical well-being, whereas the frustration or deprivation of these needs is linked to diminished functioning and increased psychological distress [8,9]. In particular, recent findings highlight that unmet psychological needs are associated with poorer mental health outcomes, including reduced well-being and increased vulnerability to distress, underscoring the central role of environmental conditions in shaping individual functioning.

### 1.1. Basic Psychological Need Satisfaction

Self-Determination Theory (SDT), proposed by Deci and Ryan [10], is a macro theory of human motivation that explains the dynamics of human need, motivation, and well-being within several social contexts. The theory defines intrinsic and extrinsic sources of motivation, and its propositions focus on how social and cultural factors facilitate or impair individual well-being and quality of performance [11,12,13]. SDT [1] encompasses six interrelated mini theories. Among these is the BPNT, which suggests that individuals are naturally inclined toward personal growth, seeking learning, mastery, and meaningful relationships as part of their inherent drive for development and integration [14].

According to BPNT [1], humans thrive in environments that allow them to fulfill their basic psychological needs for autonomy (control over one’s choices and actions in a way that aligns with their true self), competence (need achieving mastery through effective interactions with others), and relatedness (forming positive relationships and feeling securely connected and respected by important individuals). The theory suggests that the degree to which these needs are fulfilled shapes the quality of motivation (intrinsic versus extrinsic), as well as individuals’ psychological well-being, effective functioning, and overall development. The three basic psychological needs are considered universal across cultures [3,15] and when they are met in a balanced way, they contribute to positive personal development [1,16]. Environments that support these needs, enabling intrinsically motivated behaviors, are linked to greater psychological well-being [15]. On the contrary, needs frustration arises when an individual encounters behavior that obstructs the fulfillment of their basic psychological needs. This often happens in environments characterized by controlling authority figures, punishment, guilt-induction, shaming, and the withdrawal of love [15].

Given the pivotal role of the basic psychological needs in children’s and adolescents’ well-being and adjustment [1], a crucial question in development is how influential figures in socialization, particularly parents, affect psychological need satisfaction and frustration [2]. Following the distinctions among the three basic psychological needs, Soenens et al. [2] proposed three analogous types of parental support as follows:

(a) autonomy support (e.g., parents acknowledge the children’s perspective, providing choice, and encouraging exploration),
(b) competence support or structure (e.g., parents offer clear expectations, adequate help, and noncritical feedback to their children), and
(c) relatedness support or involvement (e.g., parents show respect and warmth to their children).

Past research has demonstrated that when parents adopt practices that fulfill needs, such as supporting their children’s autonomy, providing structure, and being actively involved in the relationship, it is associated with greater fulfillment of adolescents’ need for autonomy, competence, and relatedness (e.g., Soenens et al. [2]). Ratelle et al. [17], with a sample of 663 adolescents surveyed over a 3-year period, supported the unique contribution of parental behaviors (autonomy support, structure, and involvement) and psychological need satisfaction for different dimensions of school adjustment. Another study, using two longitudinal intergenerational data sets, indicated that parents’ perception of their adolescent’s need satisfaction predicted their adolescent’s self-reported need satisfaction [18]. Kassis et al. [19], with a two-wave longitudinal sample (N = 713) of students from Greece, Germany, and Switzerland, showed that family support was a protective factor of students’ autonomy, competence, and relatedness during the COVID-19 pandemic, establishing their results internationally, across genders and for students with and without migration backgrounds. However, the research on SDT has primarily focused on majority populations, often overlooking how sociocultural factors shape psychological need satisfaction in minority groups [20]. In minority educational settings students must navigate bilingual education, cultural identity negotiation, and systemic constraints, which in turn may influence how they experience autonomy, competence, and relatedness. Therefore, a broader sociocultural approach is needed to examine structural barriers and cultural dimensions of psychological need satisfaction.

While much of the empirical work on BPNT has been quantitative, qualitative studies have also shed light on how need-supportive practices are experienced in family and school contexts. For example, a qualitative study, conducted by Felber Charbonneau & Camiré [21], involving eight parent–child dyads in Canada, demonstrates how in-depth interviews can capture both converging and diverging perspectives on parental involvement in sport. The findings show that parents’ behaviors generally support athletes’ basic psychological needs, while also revealing instances of needs frustration. In addition, a study conducted in China by Kaur et al. [22], involving in-depth, face-to-face interviews with 32 undergraduate and 6 postgraduate students from both private and public universities, revealed that while the three basic psychological needs are universally important, their interpretation and fulfillment are strongly influenced by cultural factors. These studies indicate that the meaning and experience of autonomy, competence, and relatedness are shaped by both parents’ narratives and children’s interpretations, underscoring the importance of a qualitative, context-sensitive approach in the present study.

While BPNT provides a robust framework for understanding universal psychological needs, its application in minority contexts remains underexplored. In such settings, the satisfaction of autonomy, competence, and relatedness may be shaped not only by interpersonal factors, but also by structural (such as limited access to educational resources and institutional support) and sociocultural (such as linguistic diversity, cultural expectations, and minority status-related pressures) constraints. Therefore, applying BPNT in this study allows for the examination of how universal psychological processes operate under context-specific conditions.

### 1.2. Understanding Context: Intersectionality as a Lens on Need Satisfaction

While the current study is primarily grounded in BPNT [1], it also acknowledges the importance of contextual and structural influences on students’ experiences of autonomy, competence, and relatedness. In this regard, the concept of intersectionality could provide a useful lens for understanding how students’ minority status, language, and cultural identity interact with broader sociocultural and institutional forces that may facilitate or hinder the satisfaction of their psychological needs.

The term intersectionality was introduced by Crenshaw [7] to explore how social identities are shaped at the intersection of various forms of disadvantages, inequality, oppression, and discrimination. She highlighted how overlapping and interconnected aspects of identity, such as gender, race, class, and sexuality, contribute to experiences of bias and marginalization, which were overlooked by legal systems that treated race and gender as separate, unrelated categories. This aspect of intersectionality is particularly relevant when considering the satisfaction of basic psychological needs, which, although grounded in a culturally universalist perspective [1], also recognizes that the satisfaction of these needs may differ across individuals and contexts [15].

Intersectionality highlights the significance of multiple identity dimensions, such as ethnicity, language, and minority status, in shaping students’ experiences as well. For example, in bilingual or minority settings, cultural identity can be both a protective factor promoting well-being and a potential source of stress for students (e.g., De Lise et al. [23] Lee [24]). Similarly, the dual-language curriculum could also provide both opportunities and challenges for psychological needs satisfaction.

Recent studies have provided evidence for the relationship between intersectionality and both education policies and parenting practices. For example, a recent systematic review conducted by Liasidou and Gregoriou [25] suggests implications for developing intersectionality-based education policies, emphasizing the need to challenge dominant cultural and professional biases, foster critical awareness among marginalized students, and integrate intersectional perspectives into decision-making processes and support systems. In addition, Gilliam [26], using an intersectional perspective and examining minority children in Danish schools, revealed various parenting strategies to engage in their children’s schooling and well-being, shaped by both socio-cultural and socio-economic factors. Taken together, these insights from intersectionality could help contextualize the implementation of BPNT in minority settings by illuminating the sociocultural constraints and supports that shape students’ ability to satisfy their basic psychological needs.

### 1.3. Minority Educational Context and Psychological Challenges in Gökçeada

Gökçeada (In Greek: Imvros) is the largest island in Türkiye, located in the north-eastern Aegean. Prior to the 1960s, the island’s population was predominantly ethnic Greek. Since then, the Island’s demographic has changed significantly, and the Greek community now constitutes a small minority. The Middle and the High School has been serving the Greek community of Gökçeada since 2015. The school is located on the north coast of the island in Tepeköy (Agridia) with a total population of around 50, comprising both the school’s teaching staff and students. Most students come from Imvriot families who moved to the island from mainland Greece after the school was established, while many of the students are the children of Greek teachers. As all minority schools in Türkiye, the curriculum is bilingual in Turkish and Greek, with English as a third language. Consequently, while some of the teaching staff are Turkish, the majority are either Greek or Turkish of Greek origin.

An essential aspect of minority education is the influence of regulatory frameworks and institutional practices on student experience (e.g., Ebabuye & Asgedom [27]). In the case of Greek minority students in Gökçeada, the school follows the Turkish educational system and assessment methods, a framework that significantly shapes how students navigate their dual identity and the extent to which they experience competence, autonomy, and relatedness. Notably, some students have transitioned from Greek schools, creating adaptation challenges due to differences in pedagogical approaches and grading systems. Additionally, students are eligible to participate in the entrance examinations for both Greek and Turkish universities. Such regulatory factors can either support or frustrate students’ psychological needs.

In this context, structural conditions may function as sources of chronic stress for students. Navigating multiple linguistic expectations, adapting to different educational systems, and maintaining cultural identity within a majority context may create ongoing psychological pressures for students [28]. Such experiences may influence students’ perceptions of competence, limit their sense of autonomy in learning, and affect their sense of belonging. In this regard, parental support can play a protective role by enhancing students’ resilience, helping them cope with linguistic, cultural, and educational challenges, and promoting a sense of stability and emotional security [29].

### 1.4. The Current Study

The current study is a qualitative exploratory investigation examining how minority students perceive parental support in satisfying their basic psychological needs (autonomy, competence, and relatedness) within the sociocultural context of the Greek-speaking minority in Gökçeada. Students in minority educational settings often face additional pressures arising from sociocultural, linguistic, and institutional factors, which can act as chronic stressors (e.g., De Lise et al. [23]; Lee, [24]). While BPNT provides a universal framework for understanding psychological needs [1,30], it does not fully capture the role of sociocultural influences in shaping how these needs are experienced.

To address this limitation, this study integrates an intersectional lens [7] into BPNT, emphasizing that the satisfaction of autonomy, competence, and relatedness is not experienced uniformly across students, but is shaped by their positioning within multiple, overlapping social identities and sociocultural contexts. Specifically, BPNT explains *what* psychological needs are essential for well-being, while intersectionality helps explain *how* these needs are differentially experienced depending on individuals’ sociocultural positioning. In this sense, intersectionality does not replace BPNT but extends it by situating psychological need satisfaction within systems of social relations, inequalities, and institutional constraints. Therefore, rather than treating identity dimensions (e.g., ethnicity, language, minority status) as isolated factors, the study examines how their intersections shape the ways in which parental support is experienced as need-supportive or need-thwarting.

Given that parents play a pivotal role in need satisfaction and frustration [2], the present study contributes to literature not by re-establishing that parental support matters, but by examining *how* family support is experienced under conditions shaped by linguistic demands, educational asymmetries, and community-level pressures. Accordingly, the study offers two related contributions. First, it provides a contextually grounded account of how autonomy, competence, and relatedness are supported, negotiated, or frustrated in everyday family life within a minority setting. Second, it extends BPNT research by showing that psychological need satisfaction is not only influenced by parental practices themselves, but also by the intersecting sociocultural and institutional conditions through which those practices are interpreted and enacted. In this sense, the study contributes a more context-sensitive understanding of need-supportive parenting in minority education. Therefore, by examining how parental support and contextual factors shape students’ needs, the present study contributes to healthcare research by highlighting key pathways through which BPNS along with family and educational environments function as social determinants of youth mental health [8,9].

## 2. Materials and Methods

### 2.1. Research Design

This study adopts a qualitative research approach to explore the subjective experiences of students and parents in Gökçeada, a unique sociocultural setting where Greek-speaking minority students receive bilingual education. In line with qualitative research principles, the aim of this study is not statistical generalization but to provide an in-depth, contextually grounded understanding of participants’ experiences; therefore, sample adequacy was evaluated based on information richness and the capacity to generate meaningful themes rather than representativeness [31]. Therefore, focus groups were preferred over individual interviews, as they facilitated collective reflection and elicited shared experiences within a close-knit minority community [32].

Data collection was conducted through semi-structured interviews with students and parents to allow for rich, in-depth narratives. The interviews were conducted in Greek language, transcribed verbatim, and subjected to thematic analysis following Braun and Clarke’s [33] six-phase framework. This method enabled the identification of patterns across participant accounts, while remaining sensitive to the sociopolitical and cultural dimensions that shape psychological need satisfaction and frustration.

To ensure analytical rigor and transparency, we employed Saldaña’s [34] multi-cycle coding strategy. The first cycle involved inductive, exploratory coding to identify meaningful segments grounded in participants’ language and perspectives. These codes were iteratively refined and organized into categories that reflected emerging patterns. In the second cycle, axial and theoretical coding were used to explore relationships among categories and align them with BPNT constructs (i.e., autonomy, competence, and relatedness) as well as contextual factors relevant to minority experiences.

Throughout the process, we engaged in regular researcher triangulation and reflexive memo writing to interrogate our interpretations and ensure trustworthiness. This iterative, abductive process allowed for an integration of theory-driven and data-driven insights, revealing how family support, minority status, and sociocultural heritage intersect to influence students’ motivational experiences. This analytic strategy provided a flexible yet rigorous means to explore both universal and culturally specific dimensions of psychological need satisfaction in minority contexts, thereby contributing to the broader understanding of well-being through a socioculturally grounded application of BPNS.

### 2.2. Participants

Participants of the study were students and parents from Greek minority community in Gökçeada. Parents’ participation was facilitated through personal invitations from teachers. The parents were selected based on the selection criterion that their child attending middle or high school. Notably, both middle and high school are four-year programs, with students aged 11–14 and 15–18, respectively. Accordingly, the students who participated were selected with parental consent and in collaboration with their teachers. The selection criteria for students included an age range of 11 to 18 years, as well as diversity in gender and academic performance level. No parent–student pairs were included in the same focus group. Parent and student focus groups were conducted separately to minimize potential power dynamics and to facilitate more open expression. No teachers or school staff were present during the discussions.

The sample consisted of 17 participants who took part in three separate focus groups: one with parents (N = 5; 3 fathers and 2 mothers), one with lower secondary school students (N = 6), and one with upper secondary school students (N = 6); each focus group with students included 3 boys and 3 girls. In line with qualitative research principles, the aim of this study is not statistical generalization but to provide an in-depth, contextually grounded understanding of participants’ experiences; therefore, sample adequacy was evaluated based on information richness and the capacity to generate meaningful themes rather than representativeness [31].

The participants were drawn from the Greek-speaking minority community of approximately 300 inhabitants, with around 50 individuals connected to the lower and upper secondary school, comprising both the school’s teaching staff and students. While the sample is small, it represents a substantial proportion of the accessible population and is sufficient for generating in-depth, contextually grounded insights within this minority community.

### 2.3. Procedure

This study was approved by the University of Crete ethics committee (65/27 March 2024). Data were collected through semi-structured focus group interviews, allowing for a flexible yet focused exploration of the research questions. The participation was voluntary. To ensure anonymity and confidentiality, participants’ names and any identifying information were not recorded, and all data were stored securely. All participants were informed that they could withdraw from the interview at any time. The in-person interviews were conducted in Gökçeada, by four researchers of the research team who were experienced in interviewing and BPSN. Each focus group was facilitated by one researcher, while a second took observational notes. These interviews were designed to elicit detailed, narrative responses from participants, enabling a deep understanding of their experiences and perspectives. To ensure consistency, all researchers were trained on the protocol, met before data collection to discuss procedures, and debriefed afterwards to align impressions. Each focus group interview took about 50–60 min, and each researcher followed the same interview protocol. The focus group format enabled spontaneous interactions, peer elaborations, and multiple speaking turns, thereby enriching the depth of the data.

### 2.4. Focus-Group Questions

The questions were developed by the research team focusing on three components of BPNS (i.e., autonomy, competence, and relatedness) and intersectionality framework (barriers and enablers to psychological need satisfaction). However, the terms “autonomy,” “competence,” and “relatedness” were not directly used with participants. Instead, they were operationalized into everyday language (e.g., “make choices”, “feel capable”, “more connected”) to ensure clarity and avoid theoretical biasing.

Focus-group questions for parents were organized into three sections. The first section focused on students’ autonomy, exploring parents’ perceptions about students’ self-direction in academic choices (In what ways do you give your child the opportunity to make choices about their schoolwork or learning? How do you manage the balance between guiding your child and leaving space for them to decide on their own?). The second section focused on students’ competence, examining parents’ perceptions about students’ ability for mastery at school (What do you do to help your child feel capable about themselves at school? How do you usually react when your child struggles or fails at something?”). The third section focused on students’ relatedness, examining parents’ perceptions about students’ relationships at school (Can you describe a situation where your child felt disconnected or unsupported at school? In what ways do you show your child that you are available to listen and support them?).Accordingly, focus-group questions for students were organized into the same three sections. The first section focused on students’ autonomy, exploring their perceptions of their own self-direction in academic choices (How often do you feel you can make your own choices about your schoolwork or studying? In what ways do you feel supported or restricted in making your own choices about your studying?). The second section addressed students’ competence, examining their perspectives on their ability to develop mastery at school (How capable do you feel about your abilities at school? What kind of support or feedback helps you feel more capable in your studies?). The third section explored students’ relatedness, investigating their experiences of social connections at school (Can you share any experiences where you felt disconnected or unsupported in the school environment? What factors make you feel more connected to your classmates and teachers?).

### 2.5. Data Analysis

The data collected from the focus groups were analyzed using thematic analysis, following the guidelines provided by Braun and Clarke [33] and drawing on SDT [1] and the Intersectionality Framework [7] to generate themes within the context of culturally anchored research [35]. As noted by Joffe [36], thematic analysis is a systematic and reliable method for analyzing meanings in naturally occurring texts, allowing researchers to derive rich insights from raw data. Data saturation was considered to have been reached through an iterative process of data collection and analysis. Specifically, no new themes or meaningful variations emerged across the three focus groups, and thematic patterns became repetitive. Continuous comparison across groups confirmed thematic redundancy. Given the relatively homogeneous and close-knit nature of the community under study, it is likely that key shared experiences and perspectives were captured within this limited number of participants.

Based on the above guidelines and frameworks, the analysis followed six phases. First, five of the members of the research team familiarized themselves with the dataset through immersion. Data were transcribed bilingually (English and Greek), and the original texts were compared with their translated versions to identify and eliminate any mistranslations. This phase was crucial for developing a deep understanding of the data. A full list of initial codes and free nodes is provided in Appendix A. In the second phase, systematic coding was conducted by the five members, identifying segments relevant to the research questions and labeling them with concise, analytically meaningful descriptions. The entire dataset was coded thoroughly, with code labels collated and relevant data segments compiled for each code. The third phase involved generating initial themes by identifying patterns of shared meaning across the dataset. The five members of the research team discussed and grouped codes that shared a core idea to create themes that addressed the research question. In the fourth phase, they reviewed the themes to ensure they matched the coded data and the dataset, making changes as needed. Disagreements between the researchers were discussed and resolved through a systematic review of the extracts and comparison with the theoretical frameworks of the study. The fifth phase focused on refining and clearly defining each theme, making sure they were distinct and built around a strong central concept. Finally, in the sixth phase, the five members integrated analytic narrative and data extracts into a coherent account that answered the research question, incorporating an introduction, method, and conclusion with significant attention to editing.

To enhance analytical rigor, particular emphasis was placed on reflexivity, transparency, and ongoing dialog among researchers throughout the coding process. Rather than relying on statistical measures of agreement, consistency was achieved through iterative discussions and careful review of the data in relation to the study’s theoretical frameworks.

Each theme was carefully defined and named, with particular attention to conceptual clarity. The final themes were organized into overarching themes and subthemes, guided by the BPNS framework, ensuring coherence in the interpretation of the data. These themes were then integrated into a structured narrative, providing a comprehensive account of the key findings. To ensure clarity in reporting, participant responses in the findings section are labeled using (P) for parents and (S) for students, followed by a number for reference.

## 3. Results

The Results section follows a three-level analytic progression. First, it presents parental practices and students’ perceptions structured around the three BPN (Section 3.1). Second, it identifies contextual barriers and facilitators that shape need satisfaction across groups (Section 3.2). Finally, it examines how minority status specifically interacts with these needs as a structural and contextual determinant (Section 3.3).

The way the results are organized makes an important contribution to the overall interpretation of the findings, as it goes beyond a simple thematic presentation of the data and instead builds a clear, multi-level analytical framework grounded in BPNT. The findings are structured around the three basic psychological needs (autonomy, competence, and relatedness) and are then developed across three interconnected levels: (a) parental practices and students’ lived experiences, (b) contextual barriers and facilitators, and (c) the specific role of minority status as a structural factor shaping these experiences. This structure makes it possible to clearly see where parents’ and students’ perspectives converge or diverge, while also moving the analysis from everyday interactions within the family to broader sociocultural and institutional conditions. In this way, the key contribution of this organization lies in transforming the results from a descriptive thematic account into a more integrated and interpretive understanding of how BPNS is shaped within a minority educational context, thereby informing preventive and school-based mental health interventions.

### 3.1. Parental Support to Satisfy Students’ Basic Psychological Needs (QR1)

Results from the focus groups were organized around the three basic psychological needs of autonomy, competence, and relatedness, in line with BPNT (see Table 1). Each overarching theme encompasses multiple themes and subthemes, illustrated with representative quotes from both parents (P) and students (S). Across the dataset, twelve distinct themes were identified: four related to Autonomy (Demandingness, Learning by Example, Space for Mistakes, Trust/Responsibility), four related to Competence (Encouragement/Reward, Comparison with Other People, Hope Creation, Space to Play), and four related to Relatedness (Implicit/Explicit Support, Explanations/Instructions, Problem Recognition/Solution, Empathy & Resources, Family Climate). The final column indicates whether the theme was generated by parents only, students only, or both groups. Notably, several themes were reported by both parents and students, indicating shared awareness of the same relational dynamics, while certain themes (e.g., Comparison with Other People; Empathy & Resources) emerged exclusively from student accounts, pointing to experiences that parents may not have fully recognized or articulated. This asymmetry in perspective is itself a meaningful finding that informs the interpretation of the results presented below.

#### 3.1.1. Autonomy Related Parental Practices and Students’ Need Satisfaction

Parents employ various strategies to support their children’s autonomy, including setting boundaries, encouraging independence, and providing opportunities for learning through mistakes. These practices aim to guide children while fostering self-reliance. For instance, one parent (P1) shared: “Now there are boundaries, and we need to find those boundaries, which should be set.” However, students sometimes view these boundaries as overly restrictive, as S1 expressed frustration: “I used to go for a walk with my friends like this. Then my dad and my mom… tell me it’s nine o’clock to go home. And I want another half hour, but they don’t let me.” Another student (S2) added: “Sometimes I feel like they don’t trust me enough to let me decide for myself.” These quotes highlight the tension between parental intentions and students’ desires for greater freedom.

Parents also support autonomy by allowing children to learn from their mistakes. As one parent (P2) explained: “I want to let him make mistakes, understand the consequences, and have it all be his own.” Students generally appreciate this approach, with S3 noting: “To let me make your mistake,” and S4 adding: “When they let me figure it out, I feel more confident in myself.” However, trust is sometimes lacking, as highlighted by a parent (P3) who admitted: “I don’t trust my child yet with the lessons and the responsibilities he has to take care of.” Students may perceive this lack of trust negatively, as S5 stated: “It feels like they think I’m not capable, and that makes me doubt myself.”

Parents also model autonomy through their own behaviors, showing children how to manage obligations while respecting individual needs. For instance, P4 shared: “We can educate them to understand that they have certain obligations… just as we respect their break, we respect their diversity.” Students often recognize these efforts, but conflicts can still arise when parental control clashes with their preferences. As one student (S6) explained: “What I want to do, she won’t let me do. It feels like I’m not allowed to make my own choices.” This dynamic underscore the importance of balancing guidance with opportunities for independent decision-making.

These accounts show that autonomy support in this context was not equivalent to unrestricted freedom. Instead, it often took the form of guided independence, in which parents attempted to balance protection and self-direction.

#### 3.1.2. Competence Related Parental Practices and Students’ Need Satisfaction

Parents nurture competence by offering encouragement, celebrating achievements, and creating opportunities for skill-building. One parent (P5) emphasized: “We try to strengthen our children’s sense of self, that you can make it, you are capable of succeeding.” Students often respond positively to such encouragement. One (S7) remarked: “When they praise me, it makes me feel like I can do even better next time,” while another (S8) shared: “It’s like they believe in me, so I start believing in myself too.” Tangible rewards, such as stickers or smiley faces, also play a role in motivating children. A student (S9) noted: “I feel happy when I get a reward because it shows I did well,” while S10 added: “Even small rewards make me want to try harder.”

Parents also create hope during setbacks, as P6 explained: “It’s okay, you’ll get it the second time.” Students value such reassurance, with S11 stating: “When my mom says it’s okay, I feel like I can try again.” and S12 reflecting: “It helps me not give up when I see they think I can improve.” However, not all parental practices positively impact competence. For instance, (S1) expressed frustration with comparisons, saying: “They compare you to people… and you feel like you’re not good enough.” Another (S3) elaborated: “When they say, ‘Look at so-and-so,’ it makes me feel like I’ll never be good enough no matter what I do.” This highlights the potential harm of such comparisons, which can undermine confidence.

Play and exploration are additional avenues for developing competence. A parent (P2) emphasized: “Let her play! May it be happy, may it be joyful.” Students echoed the importance of these opportunities, with S3 stating: “When they let me play, I feel like I learn things on my own,” and S5 adding: “It makes me feel like I can try new things without pressure.” These practices demonstrate how parents can support competence by fostering intrinsic motivation and creating safe spaces for growth.

Overall, competence was supported when parental encouragement communicated confidence without undermining self-worth. In contrast, comparison-based practices weakened competence by shifting attention from mastery to evaluation.

#### 3.1.3. Relatedness Related Parental Practices and Students’ Need Satisfaction

Parents address the need for relatedness by being present, empathetic, and responsive to their children’s needs. P3 shared: “I’m mainly trying to be as close to her as possible, and she needs it.” Students value this presence, with S5 stating: “When I want to do something, that’s there for me no matter what I do,” and S6 noting: “It’s like knowing they’re always there makes me feel secure.” Such support fosters a sense of security and belonging, which are essential for relatedness satisfaction.

Problem-solving and guidance also strengthen the parent–child connection. One parent (P4) explained: “We are always by her side so that we can… identify and solve problems.” Students often recognize this effort, with one (S7) stating: “If I don’t know something, my parents help me figure it out. It makes me feel cared for,” while S4 added: “It feels like they’re on my team when they help me solve problems.” Parents also emphasize the importance of explanations, as P5 noted: “We try to help them by explaining a problem or an exercise.” Students appreciate this approach, with one (S9) sharing: “When they explain things, I understand better, and it feels like they care about me,” and S7 stating: “It’s like they’re teaching me, not just telling me what to do.”

The family climate also plays a significant role in relatedness. One parent (P6) highlighted: “The environment we create at home greatly influences the children.” Students echoed this sentiment, with S10 sharing: “When everyone at home is happy, I feel more comfortable talking about my problems,” and another (S12) explaining: “It’s easier to feel close to them when there’s no stress or fighting.” However, some students noted challenges in empathy. One (S1) expressed: “They demand that you put yourself in their shoes, but they don’t do the same for you.” These experiences underline the importance of mutual understanding and open communication in satisfying relatedness.

These findings indicate that relatedness was strengthened through emotional availability, collaborative problem-solving, and a supportive family climate. At the same time, relatedness was weakened when empathy was experienced as one-sided or when support became overly instructional. Thus, relatedness in this context depended on mutual recognition rather than parental presence alone.

### 3.2. Barriers and Facilitators of Students’ Basic Psychological Needs Satisfaction (QR2)

Results from the focus groups regarding barriers and facilitators of students’ basic psychological need satisfaction are presented in Table 2. Guided by an intersectional lens [6,7], the analysis considers how these barriers and facilitators are shaped by the interplay of students’ sociocultural positions (e.g., minority status, language background, and community context), rather than reflecting uniform experiences across participants. The themes reflect participants’ interpretations of everyday experiences (e.g., making choices, feeling capable, feeling connected), which were used to operationalize the core dimensions of autonomy, competence, and relatedness. The frequency values reported in the table indicate how often each theme (barriers and facilitators) was identified across the three focus groups (e.g., 1/3 = one group; 2/3 = two groups), along with the specific groups that reported each theme, as shown in parentheses (P = Parents; S-M = Middle School students; S-H = High School students).

#### 3.2.1. Barriers and Facilitators Related to Autonomy

Barriers to students’ autonomy often stem from overloaded schedules and a lack of trust from parents. Extensive academic schedules in bilingual minority school settings reduce opportunities for independent learning and self-management. One parent (P1) explained: “After 10 h of school, the child is tired. We place too much burden on them as duties.” Additionally, some parents struggle to trust their children’s ability to manage responsibilities, as illustrated by one parent’s (P2) admission: “I don’t trust my child yet with the lessons and responsibilities.” This hesitation can limit children’s ability to make decisions and feel capable of handling their own tasks. Notably, the experience of parental control as a barrier to autonomy was not confined to parents’ self-reports; it was also raised by middle school students (S-M), who described feeling restricted in their daily choices and doubting their own capacities when trust was withheld (S2, S5, S6). This barrier was reported by both parents and middle school students (2/3 groups).

However, parents also identified facilitators that enhance autonomy. Encouraging children to take responsibility and allowing them to learn from mistakes were frequently mentioned strategies. P4 shared: “I’ve given her the responsibility to organize her bag and take care of her own schedule,” highlighting how autonomy can be supported through everyday activities. Another (P3) explained: “I encourage him to make mistakes and understand the consequences,” emphasizing the importance of experiential learning in fostering independence. These facilitators were also recognized by middle school students (S-M), who reported feeling more confident and self-directed when parents allowed space for mistakes and gradually transferred responsibility to them (S3, S4). Similarly, the value of gradual independence in decision-making was articulated by both parents and S-M students, indicating that this facilitative dynamic was mutually perceived across two focus groups.

#### 3.2.2. Barriers and Facilitators Related to Competence

Barriers to competence primarily arise from resource limitations, bullying, and a lack of tailored support. Parents noted the absence of adequate educational materials, which leaves students feeling unprepared and unsupported. As one parent (P5) stated: “We don’t have books or resources, which makes it difficult for students to learn effectively.” Additionally, negative peer interactions, such as bullying, undermine students’ confidence in their abilities. P6 recounted: “She faced bullying, and it made her question her abilities,” underscoring the detrimental impact of hostile social dynamics on competence. Importantly, the barrier of bullying and social comparison was not reported by parents alone; middle school students (S-M) independently raised it as a significant source of competence frustration (S1, S3). S1 expressed: “They compare you to people… and you feel like you’re not good enough,” while S3 added that such comparisons made any effort feel futile.

Despite these barriers, several facilitators help boost students’ competence. Positive reinforcement, such as praise and rewards, plays a significant role in boosting confidence. P1 explained: “We strengthen our children’s sense of self by saying, ‘Well done, you deserve what you achieved.” Structured routines also contribute to a sense of competence, with P2 sharing: “We created a list of daily tasks, and now she follows it automatically.” These strategies provide students with clear guidance and recognition, helping them feel capable and motivated. Parental encouragement and positive reinforcement were also recognized as facilitative by high school students (S-H), who reported that praise from parents strengthened their belief in their own abilities (S7, S8, S9, S10). In contrast, play and exploration as a means of developing competence was a theme that emerged from parents and middle school students (S-M) specifically (S3, S5).

#### 3.2.3. Barriers and Facilitators Related to Relatedness

Barriers to relatedness often stem from cultural and linguistic gaps, as well as conflicts within the close-knit minority community. The diverse linguistic background of students creates challenges in forming connections. One parent (P3) noted: “Some children’s mother tongue isn’t Greek, and they struggle to communicate effectively.” Additionally, the small community size can sometimes exacerbate conflicts, with P4explaining: “The parents join the dance, and their children react negatively toward each other.” These dynamics can hinder students’ ability to build meaningful relationships. The barrier of limited opportunities for broader social connections was identified across two groups: parents and middle school students (S-M). S1 expressed feeling that empathy was asymmetrical (expected from students but not reciprocated by adults).

On the other hand, facilitators such as parental presence and a positive home environment significantly enhance relatedness. Being available and supportive fosters a sense of belonging, as P5 shared: “I try to be as close to her as possible, especially when she needs help with her studies”. Similarly, a harmonious family atmosphere strengthens connections, with P6 stating: “The environment we create at home greatly influences the children”. These practices help students feel valued and supported, both at home and within the community. Parental presence and emotional availability as a facilitator of relatedness was confirmed across parents and middle school students (S-M), with S5 and S6 describing the security that comes from knowing their parents are consistently available. This facilitator was identified across 3/3 groups (P, S-M, S-H), as middle school students (through S4’s account of collaborative problem-solving). Supportive family climate, by contrast, was a theme shared between parents and high school students (S-H): S10 and S12 reflected on how a harmonious home atmosphere made it easier to open up about personal difficulties.

### 3.3. Students’ Basic Psychological Needs Satisfaction or Frustration Due to the Minority Status (QR3)

Results from focus groups, regarding students’ basic psychological needs satisfaction or frustration due to the minority status, are presented in Table 3. Moving beyond the identification of general barriers and facilitators (RQ2), this analysis focuses on how minority status shapes students’ experiences of autonomy, competence, and relatedness. This distinction clarifies the analytical progression from identifying factors (RQ2) to interpreting them through an intersectional lens (RQ3).

#### 3.3.1. Minority Status and Autonomy

Although the results focus heavily on parental practices, they implicitly touch on the influence of the minority status of Greek students in Gökçeada. Students’ autonomy is influenced by the cultural and institutional constraints of being part of a minority group. For instance, parents noted the difficulty students face in reconciling the expectations of a bilingual education system. A parent (P1) shared: “My child’s Turkish is not good, but neither is their Greek. Trying to enforce the Turkish language, they don’t use Greek vocabulary,” highlighting the challenges students encounter in mastering two languages simultaneously. This dual-language demand often limits their ability to make independent choices or feel in control of their learning process. In this sense, autonomy frustration did not arise only from parental restriction; it was also produced by a school context in which students had to meet multiple linguistic and curricular expectations simultaneously. Yet minority status could also support autonomy when parents responded by assigning manageable responsibilities, encouraging initiative, and helping students navigate school demands without taking control away from them. Thus, autonomy in this context was constrained structurally but could still be supported relationally.

#### 3.3.2. Minority Status and Competence

Competence development is often hindered by the lack of tailored support and the unique academic pressures faced by minority students. Minority status shaped competence through unequal access to learning resources, uncertainty about language performance, and the pressure of comparison within a demanding educational environment. Participants described a context in which students were expected to succeed across more than one linguistic system while lacking sufficient materials or tailored academic support. This combination made competence especially fragile, because students’ struggles were easily interpreted as personal inadequacy rather than as consequences of structural disadvantage. At the same time, competence could be supported when families and schools provided targeted reinforcement, additional learning tools, and emotionally supportive feedback. Parents highlighted that students frequently struggle with an overloaded curriculum, compounded by the absence of adequate resources. P2 noted: “We don’t have books or material in most courses. I am calling upon myself as a teacher to tailor lessons for all grades across 12 classes.” This lack of resources contributes to students feeling unprepared and inadequate. Bullying and peer comparison also impact students’ sense of competence. As P3 explained: “She is confused about whether she is good enough in Turkish or Greek. Instead of being helped, she became more troubled by teachers sharing their personal experiences.” Such challenges undermine students’ confidence in their abilities and create additional barriers to their academic growth.

#### 3.3.3. Minority Status and Relatedness

Minority status creates unique challenges in fostering relatedness among students. Minority status shaped relatedness through the dual character of the community: it offered proximity and belonging, but also intensified visibility, conflict, and exclusion. The small size of the minority community allowed strong family and community ties to develop, which could support students’ sense of safety and belonging. However, the same closeness could magnify interpersonal tensions and transmit conflicts across family and peer relationships. In addition, language differences and cultural positioning sometimes made it harder for students to form secure peer bonds. Accordingly, relatedness in this context depended on more than emotional closeness within the family; it also relied on whether the community and school environment fostered inclusive, stable, and non-fragmented relationships. Parents noted how small community size can be both an advantage and a disadvantage. While the close-knit nature of the community provides a sense of belonging, it also amplifies interpersonal conflicts. A parent (P4) remarked: “Because we are few, everything starts with the parents, and then it’s up to the kids. The children hear things at home and react negatively toward each other.” Additionally, the cultural and linguistic diversity within minority schools often hinders students’ ability to form meaningful connections. P5 observed: “My son’s mother tongue is Turkish, and although he speaks Greek at home, he struggles to compete with others in class.” Such dynamics can lead to feelings of isolation and exclusion, which negatively impact students’ sense of relatedness.

## 4. Discussion

This study aimed to explore how parents support students’ psychological need satisfaction in the unique sociocultural and educational context of Greek-speaking minority students in Gökçeada. The results reveal that parental support plays a crucial role in fostering students’ autonomy, competence, and relatedness, yet this process is shaped by broader sociocultural and educational factors. From a healthcare perspective, these findings suggest that parental support may function as a protective factor for students’ mental health, particularly in minority contexts where additional sociocultural stressors may be present.

### 4.1. Parental Support for Students’ Psychological Need Satisfaction

Regarding RQ1, the findings confirm that parents support students’ autonomy, competence, and relatedness through practices already recognized in BPNT-informed literature, such as granting responsibility, offering encouragement, and maintaining emotional availability [2,34]. However, the present study adds nuance by showing that these practices do not carry a fixed meaning across contexts. This is an important contribution because it suggests that need-supportive parenting in minority contexts cannot be understood only in terms of whether support is present, but must also be interpreted in relation to the sociocultural conditions under which it is enacted. This may be explained by the fact that parents in minority settings often respond not only to developmental needs, but also to contextual pressures, such as cultural preservation, linguistic demands, and perceived social vulnerability. For example, parental monitoring and boundary-setting appeared to reflect not simply controlling parenting, but concerns shaped by community vulnerability, social visibility, and the need to protect children. In this sense, practices that might be interpreted as controlling in majority contexts may be experienced as protective or necessary within minority environments, where risks are perceived as more immediate or consequential.

In this sense, the findings refine existing BPNT research by showing that autonomy support in minority contexts may be expressed through forms of guidance that are more protective, negotiated, and context-sensitive than standard models often assume. Similarly, competence support was not limited to praise or structure in the abstract; it emerged in response to concrete educational pressures, including limited materials, bilingual demands, and high performance expectations. This suggests that parental support may function compensatorily, attempting to offset structural constraints that limit students’ opportunities to experience mastery within the school context. Relatedness support also extended beyond warmth alone, functioning as emotional anchoring in a context where students navigate linguistic and cultural complexity. Such emotional anchoring may be particularly critical in minority settings, where students’ sense of belonging is not always guaranteed within the broader school environment. Thus, the contribution of the present findings lies not merely in confirming that parental support matters, but in demonstrating that the meaning, function, and perceived effects of supportive parenting are mediated by the structural realities of minority schooling.

### 4.2. Barriers and Facilitators of Students’ Need Satisfaction

Addressing RQ2, the study identified several barriers and facilitators to students’ psychological need satisfaction. In the domain of autonomy, barriers were primarily associated with restrictive parental control, lack of trust, and demanding academic schedules that limited opportunities for independent functioning; these were reported by both parents and middle school students, indicating that autonomy frustration is experienced within the parent–child relationship. This pattern may reflect a tension between parental intentions to guide and protect children and students’ developmental need for independence, a tension that may be intensified in minority contexts where uncertainty and external pressures are heightened [25]. In contrast, facilitators included parental practices that promoted responsibility, allowed space for mistakes, and supported gradual independence, with similar accounts across parents and middle school students regarding how autonomy can be supported in everyday family routines [2,3].

Regarding competence, barriers included limited access to educational resources and negative peer interactions, particularly social comparison and bullying, which were reported by both parents and middle school students. These findings suggest that competence frustration in this context is not solely an individual experience but is shaped by structural inequalities and social dynamics within the school environment [18,23]. At the same time, facilitators such as parental encouragement, positive reinforcement, and structured routines were identified, with positive reinforcement recognized across both middle and high school students, while play and exploration were emphasized by parents and middle school students, indicating variation in how competence-supportive experiences are perceived across educational levels [2,15].

In terms of relatedness, barriers were linked to linguistic diversity, interpersonal tensions within the small minority community, and limited opportunities for broader social connections, primarily described by parents but also reflected in middle school students’ accounts. This suggests that relatedness in minority settings is shaped not only by family relationships but also by the structure and size of the community, where social proximity can both facilitate and constrain interpersonal connections [4,6]. Facilitators included parental presence, emotional availability, and a supportive family environment; parental presence was reported across all participant groups, whereas a positive family climate was emphasized by parents and high school students, reflecting differences in how relatedness is experienced across groups. Overall, the findings suggest that parents should be sensitive to students’ perspectives to support them efficiently (e.g., Soenens et al. [2]).

### 4.3. The Role of Sociocultural and Educational Context in Need Satisfaction

In response to RQ3, the study highlights the significant role of the students’ sociocultural and educational context in satisfying or frustrating their psychological needs. Minority status emerges as a key condition through which these experiences are shaped. The bilingual education system is a central factor influencing autonomy, as students navigate both Greek and Turkish linguistic and cultural expectations. While bilingualism can be enriching, the pressure to meet academic demands in two languages sometimes limits students’ perceived autonomy [25,26]. Additionally, competence development is influenced by the availability of educational resources, with parents and students highlighting challenges related to limited learning materials and high academic expectations. Thus, it seems that while parental support plays a crucial role, external organizational factors also contribute to psychological need fulfillment [2,23]. Relatedness is also shaped by the sociocultural environment, as students make relationships within a close community where both support and interpersonal tensions are increased. This dual role of the community suggests that proximity can simultaneously foster belonging and intensify conflict, making relatedness a particularly dynamic and context-dependent experience [13]. Bearing in mind that other studies (e.g., Chen et al. [5]) suggest that the universality of the psychological process does not exclude the possibility that individual and cultural differences could influence how people satisfy their needs, further research could explore these variations.

### 4.4. General Discussion

**Figure 1:**
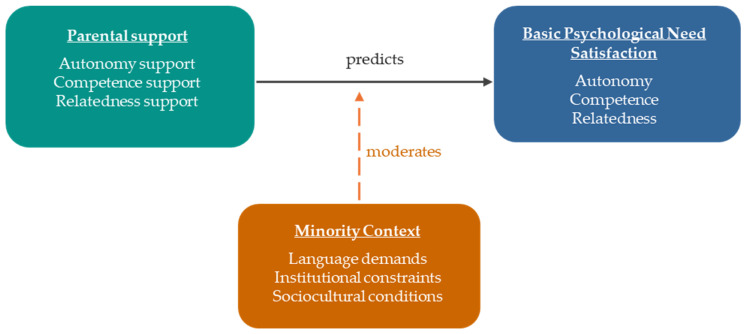
Conceptual model illustrates the relationship between parental support and BPNS, moderated by minority context.

Grounded primarily in BPNT [1], this study explored how parental support is perceived by minority students in relation to their psychological needs. By incorporating an intersectionality-informed lens [7], the study further contextualizes how minority status, language, and cultural identity may influence the expression and support of these needs. To synthesize the findings within a broader theoretical framework, Figure 1 presents a conceptual model that depicts Parental Support as the primary predictor of students’ BPNS. Critically, this pathway is moderated by Minority Context, indicating that the strength and nature of the link between parental support and need satisfaction varies depending on the sociocultural and structural conditions in which it is enacted.

As such, the coherent explanatory framework can inform future research and intervention design in minority educational settings. Given that the context is shaping parenting practices [37], the results suggest that autonomy-supportive practices need to consider the balance between guidance and freedom, especially in settings where minority status provides risks for youth populations [38]. Furthermore, the lack of educational materials and appropriate resources, which is particularly common in multilingual, under-resourced minority schools (e.g., Choi & Poudel [39]), highlights the importance of addressing structural inequalities, as essential for fostering students’ sense of competence. Finally, enhancing the need of relatedness, therefore, requires not only family support but also peer programs that promote friendship and relational inclusiveness at school settings [40].

### 4.5. Implications and Future Directions

Overall, the study contributes to BPNT by demonstrating that psychological need satisfaction [1] should be understood not only as an interpersonal process, but also as a contextually embedded phenomenon shaped by multiple identity dimensions, such as ethnicity, language, and minority status [7]. The results underscore that, while parents play a fundamental role in fostering autonomy, competence, and relatedness, their efforts are shaped by the sociocultural and educational environment which affects need satisfaction as well (e.g., Chu et al. [41]).

The results of this study have several practical implications. Schools could adopt a more integrated and context-sensitive approach by implementing structured parental training programs that promote autonomy-supportive communication, helping parents balance guidance with independence. In parallel, the provision of support services, such as language scaffolding programs and cultural facilitators, could enhance students’ sense of competence and relatedness by addressing the specific challenges of dual-language learning and small minority contexts. At the curricular level, incorporating culturally relevant materials can strengthen students’ sense of competence and relatedness [42,43]. At the relational level, targeted school-based interventions, including mentorship programs and peer support networks, and culturally competent counseling services could help students handle identity-related stressors and foster resilience [26]. These practices align with a broader healthcare perspective that emphasizes early intervention, prevention, and the promotion of mental health and well-being in school settings.

In addition, school psychologists working with minority populations can play a crucial role in facilitating collaboration between schools and families by building trust and mutual understanding. A deeper knowledge of students’ cultural backgrounds, language practices, and the historical context of minority education can help school psychologists design family-school collaboration in professional standards. This includes culturally responsive counseling, family-school partnerships tailored to minority parent’s lived experiences, and relevant efforts that address intersectionality barriers affecting psychological need satisfaction [44].

### 4.6. Limitations

While this study provides valuable insights into parental support and students’ psychological need satisfaction in the unique context of Greek-speaking minority students in Gökçeada, several limitations should be acknowledged. First, focus groups were selected as the primary method of data collection to capture shared experiences and social meaning-making processes within the community. This approach enabled participants to build upon each other’s perspectives and reflect collectively on issues related to parental support. However, it is acknowledged that focus groups may limit the expression of sensitive or dissenting views. Second, social desirability bias may have influenced participants’ responses. Parents and students may have presented their experiences in a way that aligns with social expectations rather than fully expressing negative or critical viewpoints. In small and close-knit communities, it is likely that some participants are familiar with one another, which may have influenced the openness of responses. Although some divergence in perspectives was observed, the findings should be interpreted with this limitation in mind. Third, the context-specific nature of the study means that the results may not be directly transferable to other bilingual or culturally distinct educational settings. The unique historical, linguistic, and sociocultural factors shaping the Greek-speaking minority community in Gökçeada may not be present in other educational contexts. Comparative studies with other bilingual or culturally diverse school environments from Europe or the Middle East could help determine the extent to which these results apply more broadly. Finally, the study focused primarily on parental perspectives and student experiences, without incorporating the viewpoints of teachers or school administrators. Given the significant role that teachers play in students’ psychological need satisfaction [45], relevant research could explore how teacher practices can enhance students’ autonomy, competence, and relatedness.

## 5. Conclusions

This study suggests that parental support only partially meets students’ psychological needs within the minority bilingual context of Gökçeada, because the effects of such support are shaped by broader sociocultural and institutional conditions. Parental practices such as granting responsibility, offering encouragement, and maintaining emotional closeness were filtered through the realities of bilingual schooling, limited educational resources, and the dynamics of a small minority community. Accordingly, the study contributes a more context-sensitive understanding of psychological need satisfaction by showing that minority status is not merely a backdrop to students’ development, but a structural condition that shapes *how* support is enacted and experienced. This highlights the importance of combining family-level, school-level, and policy-level interventions if students’ autonomy, competence, and relatedness are to be supported effectively in similar minority educational settings. In this way, the study advances current literature by moving beyond universalist interpretations of psychological need satisfaction and proposing a context-sensitive framework that integrates individual, relational, and structural dimensions. This contribution is particularly relevant for both educational and healthcare research concerned with promoting well-being in culturally diverse populations.

## Figures and Tables

**Table 1:** Hierarchical representation of overarching themes, themes and subthemes based on BPNS.

Overarching Theme	Theme	Subtheme/Description	Example Quotes	Theme Generator Group
Autonomy	Demandingness	Exerting control; setting boundaries on the child	“Now there are boundaries, and we need to find those boundaries, which should be set.” (P1)/“I used to go for a walk with my friends like this. Then my dad and my mom… tell me it’s nine o’clock to go home. And I want another half hour, but they don’t let me.” (S1)	Parents/ Students
	Learning by Example	Setting a good example for the child	“We can educate them to understand that they have certain obligations… just as we respect their break, we respect their diversity.” (P4)	Parents
	Space for Mistakes	Encouraging children to make mistakes; giving space to learn from them	“I want to let him make mistakes, understand the consequences, and have it all be his own.” (P2)/“When they let me figure it out, I feel more confident in myself.” (S4)	Parents/ Students
	Trust/Responsibility	Trusting the child; assigning responsibilities	“I don’t trust my child yet with the lessons and the responsibilities he has to take care of.” (P3)/“It feels like they think I’m not capable, and that makes me doubt myself.” (S5)	Parents/ Students
Competence	Encouragement/Reward	Empowering the child; rewarding efforts	“We try to strengthen our children’s sense of self, that you can make it, you are capable of succeeding.” (P5)/“When they praise me, it makes me feel like I can do even better next time.” (S7)	Parents/ Students
	Comparison with Other People	Comparing the child to others	“They compare you to people… and you feel like you’re not good enough.” (S1)	Students
	Hope Creation	Promoting optimism; reassuring children about future success	“It’s okay, you’ll get it the second time.” (P6)/“When my mom says it’s okay, I feel like I can try again.” (S11)	Parents/ Students
	Space to Play	Encouraging skill development through play	“Let her play! May it be happy, may it be joyful.” (P2)/“When they let me play, I feel like I learn things on my own.” (S3)	Parents/ Students
Relatedness	Implicit/Explicit Support	Being present; responding to needs without necessarily verbalizing support	“I’m mainly trying to be as close to her as possible, and she needs it.” (P3)/“When I want to do something, that’s there for me no matter what I do.” (S5)	Parents/ Students
	Explanations/Instructions	Providing clear explanations or guidance	“We try to help them by explaining a problem or an exercise.” (P5)/“When they explain things, I understand better, and it feels like they care about me.” (S9)	Parents/ Students
	Problem Recognition/Solution	Identifying problems (with or without child’s input) and attempting solutions	“We are always by her side so that we can… identify and solve problems.” (P4)/“If I don’t know something, my parents help me figure it out. It makes me feel cared for.” (S7)	Parents/ Students
	Empathy & Resources	Providing time, space, and emotional understanding	“They demand that you put yourself in their shoes, but they don’t do the same for you.” (S1)	Students
	Family Climate	Creating a positive, supportive home environment	“The environment we create at home greatly influences the children.” (P6)/“When everyone at home is happy, I feel more comfortable talking about my problems.” (S10)	Parents/ Students

**Table 2:** Barriers and facilitators of students’ needs satisfaction.

Basic Psychological Need	Barriers	Frequency (No. of Groups)	Facilitators	Frequency (No. of Groups)
Autonomy	Overloaded school schedules	1/3 (P)	Encouragement of responsibility	1/3 (P)
	Parental control and lack of trust	2/3 (P, S-M)	Allowing space for mistakes	2/3 (P, S-M)
	Institutional constraints of minority status (e.g., bilingual education)	1/3 (P)	Gradual independence in decision-making	2/3 (P, S-M)
Competence	Lack of educational resources	1/3 (P)	Parental encouragement and positive reinforcement	2/3 (P, S-H)
	Bullying and social comparison	2/3 (P, S-M)	Structured routines for academic success	1/3 (P)
	High academic expectations without adequate support	1/3 (P)	Play and exploration to develop skills	2/3 (P, S-M)
Relatedness	Cultural and linguistic barriers	1/3 (P)	Parental presence and emotional availability	3/3 (P, S-M, S-H)
	Small community conflicts affecting peer relationships	1/3 (P)	Supportive family climate	2/3 (P, S-H)
	Limited opportunities for broader social connections	2/3 (P, S-M)	Community engagement fostering inclusion	1/3 (P)

**Table 3:** Influence of minority status on students’ BPNS.

Basic Psychological Needs	Frustration Due to Minority Status	Satisfaction Due to Minority Status
Autonomy	Cultural and institutional constraints of minority status	Parental support for taking responsibility
	Difficulty managing the demands of a bilingual education system	Encouragement of student initiatives
	Lack of parental trust in children’s decision-making abilities	Tailored educational support for bilingual students
Competence	Lack of adequate educational resources	Targeted reinforcement of language skills
	High academic expectations without sufficient support	Supportive school environment
	Insecurity due to bilingualism and comparisons with native speakers	Provision of additional learning tools for minority students
Relatedness	Challenges in forming social bonds due to language differences	Strong family bonds
	Conflicts within the small minority community	Supportive networks within the minority community
	Social exclusion due to different cultural identity	Inclusion and friendship programs in the school environment

## Data Availability

The data presented in this study are available upon request from the corresponding author.

## Appendix A

**Table A1:** Initial Codes and Free Nodes.

Theme	Initial Codes/Free Nodes
Autonomy	Rules and boundaries set by parents; Negotiating limits; Trust and responsibility; Making mistakes and learning from them; Desire for independence; Parental overprotection
Competence	Encouragement and praise; Tangible rewards; Social comparison with peers/siblings; Experiences of failure and recovery; Structured routines and guidance; Opportunities for play and exploration
Relatedness	Parental presence and availability; Emotional support and empathy; Problem-solving together; Explanations and instructions; Positive family climate; Conflicts or lack of empathy
